# Involvement of ELAV RNA-binding proteins in the post-transcriptional regulation of HO-1

**DOI:** 10.3389/fncel.2014.00459

**Published:** 2015-01-15

**Authors:** Marialaura Amadio, Giovanni Scapagnini, Sergio Davinelli, Vittorio Calabrese, Stefano Govoni, Alessia Pascale

**Affiliations:** ^1^Department of Drug Sciences, Section of Pharmacology, University of PaviaPavia, Italy; ^2^Department of Medicine and Health Sciences, University of MoliseCampobasso, Italy; ^3^Inter-University Consortium “SannioTech”Benevento, Italy; ^4^Department of Biomedical Sciences, University of CataniaCatania, Italy

**Keywords:** heme oxygenase-1, ELAV, hemin, post-transcriptional regulation

## Abstract

Heme oxygenase-1 (HO-1) is an inducible rate-controlling enzyme of heme catabolism. The cytoprotective function of HO-1 activity has been verified in multiple studies, and together with its by-products is considered a key component of the cellular stress response. The transcriptional induction of HO-1 has been largely studied in response to multiple forms of stressful stimuli but our understanding of HO-1 post-transcriptional control mechanisms in neuronal cells is currently lacking. In the present report we show the involvement of the RNA-binding proteins (RBPs) embryonic lethal abnormal vision (ELAV) in the regulation of HO-1 gene expression. Our study demonstrates a specific binding between HO-1 messenger RNA (mRNA) and ELAV proteins, accompanied by an increased expression of HO-1 at protein level, in a human neuroblastoma cell line treated with hemin. Clarifying the induction of HO-1 expression at post-transcriptional level may open therapeutic perspectives for treatments associated with the modulation of HO-1 expression.

## Introduction

Heme oxygenase-1 (HO-1) is an intracellular enzyme that catalyzes the initial and rate-limiting step in the oxidative degradation of heme, and generates biliverdin, free iron (Fe^2+^), and carbon monoxide (CO; Calabrese et al., 2006). HO-1 is a heat shock protein (HSP32) and it is recognized as one of the major stress-inducible protein in mammalian cells (Maines, 1997). HO-1 activity can increase several-fold in response to a wide variety of stimuli that cause changes in the cellular redox state (Ryter and Choi, 2002). The products of heme metabolism such as bilirubin, ferritin and CO, mediate many of the anti-inflammatory and antioxidant effects associated with the potent cytoprotection provided by HO-1 enzymatic system (Kirkby and Adin, 2006). HO-1 expression is induced ubiquitously in response to oxidative challenges but brain tissue is particularly susceptible to free radical damage (Poon et al., 2004). Indeed, there is an increasing support that HO-1 plays a crucial protective role in the central nervous system (CNS), primarily in astrocytes and microglia/macrophages, particularly during aging and in several disease states where oxidative stress is implicated, including Alzheimer’s disease (AD), Parkinson’s disease (PD) and Huntington’s disease (HD; Calabrese et al., 2004). Although numerous published reports have demonstrated multiple beneficial effects of HO-1, its mechanism of action has not been completely elucidated. Expression of HO-1 is regulated essentially at transcriptional level, even though a post-transcriptional modulation of HO-1 messenger RNA (mRNA) in distinct cellular contexts has been described (Gozzelino et al., 2010). For instance, in human dermal fibroblasts hypoxia regulates HO-1 gene expression by a specific post-transcriptional mechanism: stabilization of mRNA (Kitamuro et al., 2003). Therefore, the regulation of HO-1 mRNA levels in response to cellular stress may be induced by both transcriptional and post-transcriptional events that act independently, and vary in function of the stress inducer (Leautaud and Demple, 2007). Although post-transcriptional control mechanisms are not yet fully understood, it is clear that they are key determinants in the regulation of mRNA stability and translation (Bolognani and Perrone-Bizzozero, 2008; Pascale et al., 2008; Keene, 2010). In particular, RNA-binding proteins (RBPs) regulate gene expression at post-transcriptional level and influence pre-mRNA processing as well as transport, localization, stability and translation of target mRNAs (Dreyfuss et al., 2002). RBPs are crucial in many aspects of cellular physiology and may play a direct role in the pathophysiology of neurodegenerative diseases (Pascale and Govoni, 2012; Perrone-Bizzozero and Bird, 2013; Romano and Buratti, 2013). Recent studies have identified hundreds of RBPs previously unknown (Castello et al., 2012) and a new and fascinating idea is that neurons have their own systems for regulating RNA metabolism, processing, localization, and expression (Darnell, 2013). In this context, embryonic lethal abnormal vision (ELAV) proteins are RBPs mostly expressed in neurons and post-transcriptional regulation in neuronal cells strongly depends on the control exerted by these RBPs (Pascale et al., 2008). In vertebrates, the ELAV (or Hu) family comprises the neuron-specific members HuB, HuC and HuD, and the ubiquitously expressed HuR (Colombrita et al., 2013). Moreover, emerging insights into the regulation by which cells establish patterns of gene expression suggest that post-transcriptional events influence inducible genes and signaling cascades such as HSPs and oxidative stress-activated pathways (Abdelmohsen et al., 2008; Amadio et al., 2008). HO-1 gene product and its mRNA abundance is controlled at various stages and it was reported that HO-1 induction by nitric oxide (NO) is regulated by the HuR/ELAV. Interestingly, the HuR/ELAV was found to associate with HO-1 mRNA, and this interaction increased following NO treatment (Kuwano et al., 2009). The HO-1 chemical inducer hemin is a highly reactive compound exhibiting pro-oxidant properties in several biochemical reactions. In the present study, we investigated, in SH-SY5Y human neuroblastoma cells, the expression of HO-1 mRNA and protein following hemin exposure and whether in these conditions HO-1 mRNA may represent a target of ELAV RBPs.

## Materials and methods

### Cell cultures and treatments

The SH-SY5Y human neuroblastoma cells were grown in Eagle’s minimum essential medium supplemented with 10% fetal calf serum, 1% penicillin-streptomycin, L-glutamine (2 mM), nonessential amino acids (1 mM), and sodium pyruvate (1 mM) at 37°C in an atmosphere of 5% CO_2_ and 95% humidity, as previously described (Racchi et al., 2003). The cells were exposed to the solvent (PBS) or to hemin (50 or 100 µM as reported in figure legends) for 2 h.

### Reverse-transcription and real-time quantitative polymerase chain reaction (qPCR)

Total RNA was extracted from cells using the RNeasy Micro Kit (Qiagen, Milan, Italy). The reverse transcription was performed following standard procedures. PCR amplifications were performed using the LightCycler instrument (Roche Molecular Biochemicals) in the presence of QuantiTect SYBR Green PCR mix (Qiagen, Milan, Italy), with primers designed by using the PRIMER3 software.^1^ Primer sequences were as follows: HO-1, 5′-AGC AAC AAA GTG CAA GAT TCT GC-3′ (forward); 5′-CAG CAT GCC TGC ATT CAC ATG-3′ (reverse); product size: 161 bp; RPL6 (Ribosomal Protein L6), 5′- AGA TTA CGG AGC AGC GCA AGA TTG-3′ (forward), 5′-GCA AAC ACA GAT CGC AGG TAG CCC-3′ (reverse). RPL6 mRNA was chosen as the reference mRNA on which HO-1 was normalized because this RNA remained substantially stable during the treatments. Since RPL6 mRNA does not bear ARE sequences, it was also used as negative control in the immunoprecipitation experiments coupled with real-time quantitative polymerase chain reaction (qPCR).

### Immunoprecipitation

Following treatment, SH-SY5Y cells were harvested and homogenized in a buffer [containing 20 mM Tris-HCl (pH 7.4), 2 mM EDTA, 0.5 mM EGTA, 50 mM 2-mercaptoethanol, 0.32 mM sucrose, and a protease inhibitor cocktail] by using a teflon/glass homogenizer. Immunoprecipitation was performed on total homogenates according to a previously published protocol (Amadio et al., 2009). Briefly, immunoprecipitation was carried out at room temperature for 2 h using 1 µg of anti-ELAV antibody (Santa Cruz Biotech, CA, USA) per 50 µg of proteins diluted with an equal volume of 2× Immunoprecipitation Buffer [2% Triton X-100, 30 mM NaCl, 20 mM Tris-HCl (pH 7.4), 2 mM EDTA, 2 mM EGTA, 0.4 mM sodium vanadate, protease inhibitor cocktail and a RNAase inhibitor] in presence of 50 µl of protein A/G plus agarose beads (Santa Cruz Biotech, CA, USA) previously blocked with 5% BSA in PBS. The samples were finally subjected to RNA extraction and reverse transcription. The negative control was obtained in the same conditions, but in presence of an irrelevant antibody with the same isotype of the specific immunoprecipitating antibody. For binding assay, 100 µl of the immunoprecipitation mixes were collected from each sample and used as “input signal” to normalize the real-time qPCR data. For both HO-1 and RPL6, the mRNA content present in the immunoprecipitated pellet has been normalized on the mRNA amount present in the relative “input signal”.

### Western blotting

Total lysate were diluted in 2× sodium dodecyl sulfate (SDS) protein gel loading solution, boiled for 5 min, separated by 12% SDS-polyacrylamide gel electrophoresis, and then processed following standard procedures. The antibodies anti-ELAV, anti-HO-1, and anti-α-tubulin (Santa Cruz Biotechnology, Santa Cruz, CA) were diluted in TBST buffer [10 mM Tris-HCl, 100 mM NaCl, 0.1% (v/v) Tween 20, pH 7.5] containing 5% milk. The nitrocellulose membranes were processed with Pierce ECL Plus from Thermo Scientific (Rockford, IL, USA). The experiments were performed at least on three different cell preparations using α-tubulin to normalize data. Densitometric analysis was performed using the NIH Image software.^2^

### Data analysis

For statistical analysis the GraphPad Instat statistical package (version 3.05 GraphPad software, San Diego, CA, USA) was used. The data were analyzed by analysis of variance (ANOVA) followed, when significant, by an appropriate *post hoc* comparison test, as indicated in figure legends. Differences were considered statistically significant when *p* values ≤ 0.05.

## Results

### Hemin increases HO-1 mRNA levels in SH-SY5Y cells

**Figure 1:**
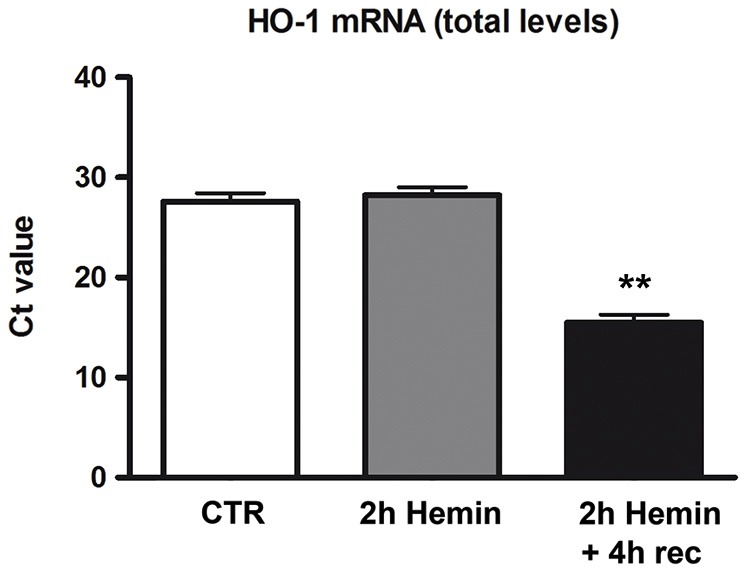
**Effect of hemin exposure on HO-1 mRNA levels**. Determination of HO-1 mRNA levels by real-time qPCR in human neuroblastoma SH-SY5Y cells following treatments with 100 µM hemin for 2 h + 4 h recovery (rec). HO-1 mRNA contents are expressed as means of the cycle threshold (Ct) ± S.E.M. **p < 0.01, Dunnett Multiple Comparisons Test, *n* = 5.

To determine whether hemin treatment affects HO-1 expression in human neuroblastoma SH-SY5Y cells, we first measured HO-1 mRNA levels in total homogenates of cells treated with hemin (100 µM) for 2 h + 4 h recovery. Real-time qPCR data demonstrated a marked increase of HO-1 mRNA level in 2 h hemin-treated samples followed by 4 h recovery as compared to the other samples (Figure 1). RPL6 mRNA level remained substantially stable during the experiments (data not shown). These data reveal that in SH-SY5Y cells hemin treatment up-regulates the expression of HO-1 mRNA.

### Association of HO-1 mRNA with ELAV RBPs

**Figure 2:**
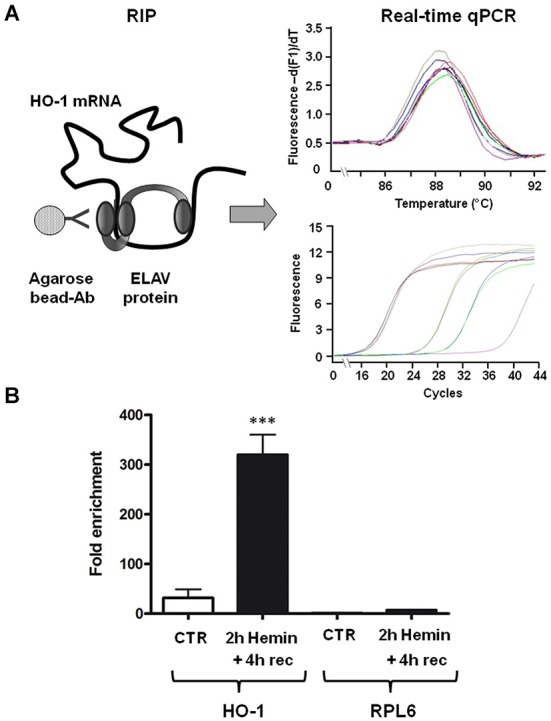
**ELAV proteins specifically bind to HO-1 mRNA. (A)** Schematic representation of the procedure of the RNA-immunoprecipitation (RIP) coupled with the real-time qPCR to determine the interaction between ELAV proteins and HO-1 mRNA (left) and representative real time qPCR melting curves with the corresponding amplification plots (right). Ab: antibody **(B)** Fold enrichment detected by real-time qPCR of HO-1 and RPL6 (housekeeping) mRNAs in control (CTR) and 2 h hemin-treated + 4 h recovery (rec) SH-SY5Ycells following immunoprecipitation with the anti-ELAV antibody. ***p < 0.001, Tukey-Kramer Multiple comparisons test, *n* = 3.

To test the hypothesis that ELAV RBPs interact with HO-1 mRNA following hemin treatment, we performed an immunoprecipitation assay on SH-SY5Y cells, which express all the four isoforms of ELAV proteins, followed by real-time qPCR. As shown in Figure 2, the association of ELAV RBPs with HO-1 mRNA occurs; indeed, we found that the amount of HO-1 transcript bound by ELAV proteins in the total homogenate is increased following 2 h hemin stimulus + 4 h recovery (Figure 2). Moreover, the housekeeping RPL6 mRNA was almost undetectable in the same immunoprecipitated pellets (Figure 2), confirming the existence of a specific binding between ELAV proteins and HO-1 mRNA.

### Hemin treatment upregulates HO-1 protein levels in a concentration-dependent manner

**Figure 3:**
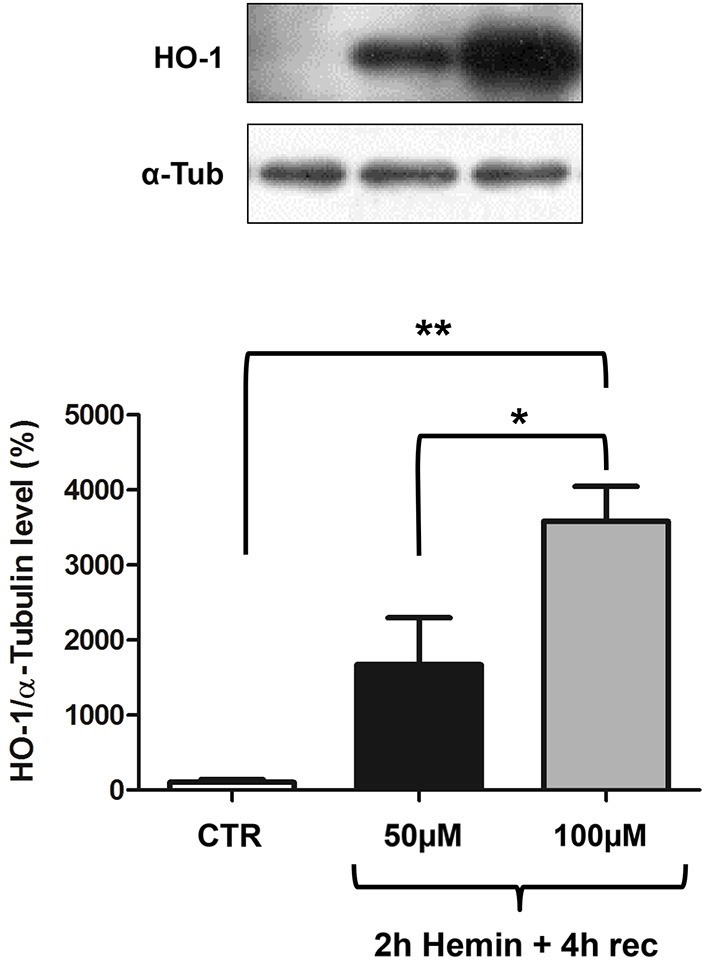
**Hemin treatment affects HO-1 protein total levels in SH-SY5Y human neuroblastoma. (Upper)** Representative Western blotting of HO-1 and α-tubulin in control (CTR) and hemin-treated SHSY5Y cells. Cells were exposed to either 50 or 100 µM hemin for 2 h followed by 4 h recovery (rec). **(Lower)** Mean gray levels ratios (mean ± S.E.M.) expressed as percentages with respect to control (100%) of HO-1/α-tubulin immunoreactivities measured by Western blotting. *p < 0.05; **p < 0.005, Tukey-Kramer Multiple comparisons test, *n* = 3.

We finally measured HO-1 protein levels by Western blotting, finding an increase after 2 h hemin exposure followed by 4 h recovery (Figure 3). Interestingly, in basal conditions, HO-1 protein content is almost undetectable and its increase is proportional to hemin concentration, reaching statistical significance only at 100 µM concentration (Figure 3). In the same samples we also measured ELAV proteins levels, finding they are not modified following hemin stimulus (not shown).

## Discussion

HO-1 is one of the main players of the endogenous antioxidant responses and since it provides cytoprotection against various neurotoxic insults, it is crucial to elucidate how HO-1 gene expression is regulated. A comprehensive knowledge regarding HO-1 expression in human neural cells is still lacking. Although most studies have focused on the molecular mechanisms responsible for the cytoprotective effects of HO-1, the importance of post-transcriptional processes in the modulation of HO-1 gene expression has been greatly underestimated. Furthermore, there is an emerging consensus that neuronal transcripts are differentially processed in the brain than in other tissues (Darnell, 2013). Interestingly, it was recently demonstrated that RBPs and non-coding RNAs are critical components underlying the post-transcriptional mechanisms for the coordinate regulation of mRNA expression in neuronal systems (Loya et al., 2010). Human ELAV RBPs are involved in the post-transcriptional control of several early-responsive genes such as MYC, FOS and cytokines (Keene, 2007; Papadopoulou et al., 2013). Since HO-1 system is a promising approach to treat specific disorders and its induction has significant consequences in the CNS, the present study explores the role of ELAV proteins in the regulation of HO-1 expression at both mRNA and protein levels. In particular, we treated human neuroblastoma SH-SY5Y cells with hemin, a pro-oxidant molecule. Although HO-1 is activated by various electrophilic compounds including polyphenols (Davinelli et al., 2013), hemin is one of the most effective stressors in terms of reactive oxygen species (ROS) production and it is widely used to study HO-1 function. Therefore, we chose hemin as a challenge for our experiments. However, it is essential to point out that at elevated concentrations hemin may contribute to cell injury by cytotoxic effects, although strengthening endogenous defense against oxidative stress, at nontoxic concentrations its therapeutic potential has been reported in multiple acute injury models, including those at brain level (Lu et al., 2014). In our study, we found that HO-1 expression is induced in 100 µM hemin-treated neuronal cells, as demonstrated by real time qPCR and Western blotting experiments (Figures 1, 3). This finding is consistent with previous studies showing the activation of HO-1 by hemin in human neuroblastoma cells (Nakaso et al., 2003). Interestingly, and in accordance with previous evidence in rat hippocampal neurons (Scapagnini et al., 2004), we observed that, in human SH-SY5Y neuroblastoma cells, HO-1 protein expression is almost absent in basal conditions and it is strongly induced by hemin in a concentration-dependent manner (Figure 3). However, we cannot exclude that the sensitivity of Western blotting technique is not sufficient to detect very low amount of protein as in control cells. The hemin-induced increase of HO-1 protein may be due to a positive regulation of HO-1 at post-transcriptional level. Indeed, our results shows a specific association between ELAV and HO-1 mRNA (Figure 2), suggesting that this binding may have potential consequences for HO-1 protein expression. Our hypothesis is that HO-1 is present as mRNA in control cells so that, as many early response genes, it can be rapidly translated following a specific stress stimulus, such as hemin exposure, thus contributing to the physiological cellular response.

**Figure 4:**
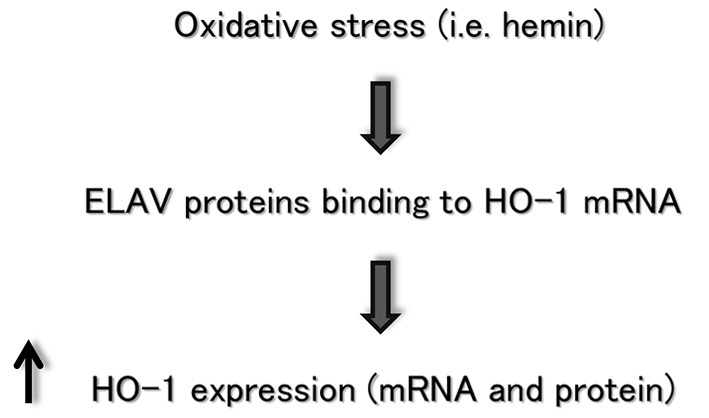
**Flow chart depicting the ELAV/HO-1 cascade, in which an oxidative stress stimulus favors ELAV proteins binding to HO-1 mRNA, producing an increase of HO-1 expression at post-transcriptional level**. See the text for more details.

Although it was well-established that HO-1 is highly inducible by a large number of stressful stimuli such as heme or certain other metalloporphyrins, the regulation of gene expression and induction of HO-1 is complicated by the fact that its regulatory response is not restricted to heme or other physical and chemical factors. A purpose of this study was to bridge the significant gap in understanding the regulation of HO-1 induction in neural cells. Previous evidence from our group showed that the ELAV RBPs are involved in regulating the post-transcriptional fate of HSP70 and SOD-1 mRNA following H_2_O_2_-mediated oxidative stress in SH-SY5Y cells (Amadio et al., 2008; Milani et al., 2013). HSP70, SOD-1 and HO-1 modulate crucial defensive mechanisms for neurons exposed to an oxidant challenge. Consistently with the data reported for the other genes, the results described in this report suggest a novel role for ELAV RBPs in regulating, post-transcriptionally, HO-1 expression in SH-SY5Y neuronal cells, as schematically represented in Figure 4. Future studies are needed to identify whether this interaction is a characteristic feature of HO-1 induction during the cellular stress response.

## Conflict of interest statement

The authors declare that the research was conducted in the absence of any commercial or financial relationships that could be construed as a potential conflict of interest.

## Footnotes

^1^http://bioinfo.ut.ee/primer3-0.4.0/primer3/input.htm

^2^http://rsb.info.nih.gov/nih-image
